# Notes on a Case of Fatal Poisoning by Metallic Arsenic

**Published:** 1850-01

**Authors:** B. Silliman

**Affiliations:** Professor of Chemistry and Toxicology in the University of Louisville, and of Chemistry applied to the Arts, in Yale College; Louisville, Ky.


Art. IV.—*Notes on a Case of Fatal Poisoning by Metallic Arsenic* By B. Silliman, Jr., M. D., Professor of Chemistry and Toxicology in the University of Louisville, and of Chemistry applied to the Arts, in Yale College.

The case here related is of interest chiefly because of the comparative rarity of this mode of arsenical poisoning. The facts of the case and the steps of my examintion are given very nearly in the words of the deposition—this may account for the want of condensation in this communication, which might otherwise be presented in much fewer words. It may not, however, be without interest to the general reader, as showing, to some degree, the manner in which the toxicologist proceeds in such cases.

---

*Works of Dr. Wells, p. 49.

On the fourth day of September, 1849, Dr. J. W. C., of Bristol, Connecticut, brought to my laboratory the stomach of a man, who, as he stated, died in the town of Bristol, under suspicious circumstances, leading to the supposition that he had been poisoned. I consented to make a toxicological examination of the stomach, at his request. This I commenced in his presence immediately after the presentation. The stomach was presented to me in a vessel of alcohol; both the orifices of the organ were tied with ligatures, which, as I was informed, were put on before its removal from the body. The stomach had not been opened; it was to appearance fresh, no change nor decomposition having taken place; nevertheless it appeared of a livid and unnatural color. A strong, horizontal line divided it into two equal portions, the lower portion being dark, the upper opaque and not unnatural in its appearance. With Dr. C.'s assistance I opened the stomach by a long incision, exposing its interior. Its internal appearance was strikingly unhealthy, livid and inflamed, resembling the effects of acute gastritis. Its contents appeared singularly unnatural, being not less in quantity than one pint of dark colored, brownish fluid, thick, and resembling in color and appearance rich chocolate. The line of division spoken of before, was not so apparent on the interior as on the exterior. The whole interior surface presented strong evidence of inflammation; the color was livid, with lines of extravasated blood. The mucous coat was lined with the chocolate colored matter before mentioned. I detected no trace of food in the stomach, save a few filaments of reddish matter resembling the skins of tomatoes.

My first chemical examination was made upon the contents of the stomach. I proceeded to analyze the chocolate colored matter. After reducing it to a transparent solution by means of agents of known purity, I submitted it to the action of a current of sulphuretted hydrogen. I was soon satisfied, from the orange colored precipitate that was produced, that there was some metallic substance present in the stomach. This precipitate was not very abundant, nor of so deep an orange color as to be of the most decided character. It was, however, completely soluble in caustic ammonia—a character which belongs to the sulphuret of arsenic. The evidence obtained by this preliminary trial was such as to induce farther and more critical examination as to whether the suspected substance was arsenic, antimony, cadmium, or tin—the only metals capable of producing a yellow compound with sulphur under the circumstances above described. I searched, at first, in vain upon the coats of the stomach for anything resembling a white powder.

My next step was to take a portion of the substance of the stomach itself, as well as of its contents. This I proceeded to reduce, by means of the process of Fresenius and Von Babo, to the state of a perfect and colorless solution. This process, occupying several consecutive hours, was conducted with care, to avoid contamination from every source—meanwhile, with the advantage of a stronger light, I employed myself with a more critical optical examination of the surface of the coats of the stomach, hoping to detect thereby something which should indicate the probable cause of the appearance before described. On scraping the dark brownish matter from off the coats of the stomach, I observed in several places, adhering firmly to the mucous coat of the organ, dark colored grains, resembling at first sight grains of black pepper; some of them, however, had a metallic lustre and resembled tinfoil. On inspecting these metallic grains, it at once occurred to me that they might be metallic arsenic, or "cobalt," as this substance is absurdly called, in the shops of the apothecary and in commerce. I accordingly proceeded to determine by experiment the true nature of these grains. On heating some of them in a closed tube of glass they were entirely volatilized, or sublimed, lining the interior of the tube with a brilliant metallic coat, whose surface reflected like a mirror. The extreme borders of this metallic ring were fringed with a white crystaline powder. Another portion of these grains was in like manner volatilized, but with the access of air. In this case the dark metallic coat at first formed was rapidly changed to a white crystaline coating, which on examination with a magnifying glass was seen to consist of a great number of minute but very brilliant eight-sided figures. Another tube, in which a portion of these metallic grains from the stomach had been sublimed and converted by the aid of heat and air, into the white crystalline lining last described, was next treated by a current of sulphuretted hydrogen gas, by which means, with the aid of heat, the white coating was completely converted into a brilliant yellow substance, entirely resembling the yellow sesqui-sulphuret of arsenic, or orpiment. That no doubt might remain as to the real nature of this substance, I proceeded next to pass through the same tube a current of strong ammoniacal gas. The yellow coating was thereby immediately dissolved into a clear transparent yellow fluid. By the aid of a gentle heat the ammonia was expelled from this fluid, and the original yellow coating reappeared as before. The series of properties here described is found in no other substance than arsenic.

The evidence above detailed is such as produced the strongest conviction that the substance under examination was metallic arsenic, and that it could be nothing else.

The above experiments were conducted mainly during the interval of two days, in which the portion of the stomach already alluded to and its contents were in process of transformation from the solid condition to that of fluidity. My next trials were made on the solution thus produced, with a view to the detection of similar evidence in it. The result, however, already obtained, led me to expect, that the evidence to be derived from the muscular tissues of the stomach, would be much less remarkable than that obtained on the solid contents, to wit: the metallic grains before spoken of. The solution was treated with a current of sulphuretted hydrogen gas. In a short time a very decided precipitate, of an orange yellow color, appeared in the solution, after standing for some hours in a warm place. This was collected upon a filter, separated from the solution, and carefully dried at a regulated temperature. A portion of this yellow precipitate was mixed with appropriate reducing agents, in a tube of hard glass, and exposed for a short time to a red heat; the neck of the vessel during this process of heating, became lined with a dark metallic-looking coating, resembling in all respects the similar rings produced by arsenic.

Another portion of this yellow precipitate produced from the solution of the stomach, was treated with caustic ammonia—complete solution ensued. This is a characteristic property of the yellow sulphuret of arsenic.

I next proceeded to try a test which is regarded by most chemists as one of peculiar certainty. This test is known as Reinseh's test; from the name of the discoverer. I regard it as of any single tests the most satisfactory, as well from the unequivocal evidenee which it yields as from the facility of its application in cases where other tests may give equivocal evidence. A portion of the suspected substance made slightly acid by hydrochloric acid, and freed from turbidness by filtration, is boiled for a few moments in contact with a slip of bright metallic copper. If arsenic is present in the most minute quantity, the surface of the copper becomes immediately tarnished, and assumes the color of steel more or less completely in proportion as the quantity of arsenic may be greater or less. This test was applied to the case in hand. The bright metallic copper, after a few moments boiling it, was placed in contact with a portion of the tissues of its contents treated as just described, and assumed distinctly the grey color of steel. A portion of this copper cut from the slip, was heated in a tube of glass, whereby the grey coating was immediately transferred from the copper to the glass, lining it with a similar metallic mirror to those which had been previously produced in the tubes before described. This coating derived from the copper, gave all the reactions which have been described as peculiar to arsenic.

It is well known to chemists, that metallic arsenic, when heated in contact with the air, burns with a peculiar odor, which is described as the garlic-like or alliaceous odor. This character is much insisted upon by some writers on medical jurisprudence, as one of great importance. I obtained the garlic like odor, as well from the metallic grains found on the coats of the stomach, as from the yellow precipitate obtained from dissolving the substance of the organ itself.

It was deemed unnecessary, after the very sufficient and convincing testimony already obtained, to resort to any of the numerous and less satisfactory means known to chemists for the detection of arsenic.

The production of the metallic ring; its entire volatility by heat; its conversion, by the aid of heat and air, into brilliant white octohedral crystals; the further transmutation of these, by means already described, into yellow orpiment; the solubility of this yellow substance in ammonia; its reproduction, unaltered, upon evaporation of the ammonia; and finally the reconversion of this yellow substance, by reducing agents, into the original brilliant metallic mirror, forms a chain of consecutive evidence of the most satisfactory character, and such as can be produced by no other substance in nature than arsenic.

I should regard the production of the metallic mirror of the alliaceous odor, and of the white crystalline grains found on heating the black mirror, a sufficient and irrefragable proof that the suspected substance was arsenic.

I am satisfied, as the result of my research in this case, that death was produced by the administration of metallic arsenic, otherwise called cobalt or fly powder.

I was unable to form any opinion as to the quantity of metallic arsenic present in the stomach.

There is evidence on record that life has been destroyed by arsenic administered in doses of from three grains to as many ounces. It would probably require a greater quantity of cobalt than of the white arsenic to destroy life, since it is only the small part of the metal, which has become partly oxydised, which acts as a poison. It is remarked by writers on medical jurisprudence, that a frequent symptom from poisoning by arsenic is the secretion by the stomach of a dark brownish chocolate-like fluid, which is not unfrequently ejected by vomiting, and which is found in the stomach and intestines on a postmortem examination.

It is a well known property of arsenic to act as a preservative of animal matter. Its antiseptic properties are so strong that in many cases of poisoning by this substance, the stomach and organs of digestion have been found in a perfect state many months and even years after interment.

The medical testimony in this case is less satisfactory than the chemical. The patient had been unwell for some days with symptoms supposed to be occasioned by simple derangement of the bowels, accompanied by nausea and vomiting. The medical attendant was called on Thursday evening, Aug. 30, and found the patient in bed. He complained of nausea, thirst, and constant distress at the stomach, and pain in his bowels ; pulse feeble and irregular ; his hands cold. He was treated with Hopkins's elixir, followed by Dover's powders, camphor gum and sudorifics. The following day he appeared easier, and was in a perspiration. Still complaining of his stomach, an emetic was administered of ℞. Antim. tart. grs. ii., pulv.

ipecac grs. xx.; which, failing to act, was repeated—each in three doses.

He was not seen again until nine o'clock on Friday night, when he was in a state of collapse, with great distress in the stomach and great difficulty of breathing; pulse not perceptible; hands and feet cold and livid. He complained of extreme heat in the pit of his stomach, and conversed with difficulty; had a constant disposition to vomit, general twitching of the muscular system, and frequent alvine discharges; countenance pallid; skin cold and bedewed with a clammy sweat. Diffusible stimulants were administered, and he died about midnight following, say thirty-six to forty hours from the time he was first seen by a medical attendant.

The autopsy detected nothing remarkable in the upper viscera. The stomach, however, showed distinct marks of inflammation, by a medial ring of vermillion red, extending around it; below—say three-fourths of the organ—was dark, nearly black, colored; the upper portion healthy. It does not appear that the alimentary canal was examined. The appearance of the stomach, when opened, has already been alluded to.

We certainly cannot fail to recognize here several of the main features of arsenical poisoning, but of a comparatively mild type. It is not stated whether there was injection of the conjunctiva, and no medical attendant was present during the three or four hours immediately preceding death. We are not able, therefore, to decide whether tetanic convulsions were developed. Nor indeed are these by any means universal in arsenical poisoning, although frequently present. The "muscular twitchings" however, recorded in the testimony, look that way. No mention is made by the medical attendant as to the nature of the matter vomited—but judging from the abundance of the dark brown tinted matter, mixed with mucus, which I found in the stomach, we cannot doubt that this matter was also ejected. This case presents, then, the the following train of symptoms, all indicative of arsenical poisoning, viz: Faintness; depression; nausea, with intense burning pain in the pit of the stomach; constant thirst; pain more intense at the close; pulse very feeble at first, and wholly imperceptible at last; diarrhea; muscular contractions, or twitchings; difficult respiration; cold and clammy skin in the collapse; and brown turbid secretion in the stomach.

It is doubtful, on a review of the symptoms, whether any characteristic symptoms is wanting; and, certainly, when taken in connection with the chemical evidence, there can be no doubt that death was occasioned in this case by metallic arsenic.

**Louisville, Ky., Dec. 19, 1849.**

